# Annotations

**Published:** 1898-01-22

**Authors:** 


### Annotations.

### Manchester Royal Infirmary.

The difficulty as to what is to be done in regard to the Manchester Royal Infirmary still continues, and probably will continue until the many interests involved can make up their minds what it is they really want. It is no doubt this diversity of interests which has caused the extraordinary delay which has occurred in carrying into effect a most necessary reform, for no one can doubt the deficiencies of the present building. It is impossible for Manchester to ignore the interests of its great college, or to put on one side the requirements of the students, however much the governors of the infirmary may object to any interference in the management of their institution. A new infirmary, on a site convenient to the patients, the students, and the professors alike, is obviously necessary. At least we should have thought so. But Manchester is a sentimental city, and cannot bear the thought of its Piccadilly being altered or encroached upon; while some people connected with the infirmary, and even some of the staff, cannot bear to see any change in the appearance of a building with which so many old memories are associated. Whatever is done, it is of the utmost consequence that the governors should recognise the overwhelming importance of a medical school in promoting the highest efficiency in the working of a large hospital.

### "Drunk or Dying?"

Another of the unfortunate cases in which a patient with fractured skull, and slowly progressive intra-cranial hæmorrhage, has been sent away from a hospital under the impression that he was only drunk, has been the subject of investigation by Dr. Ambrose, the coroner for West Ham. The deceased was found by a policeman at eleven o'clock at night lying on his back in the roadway, bleeding from a wound on the head, and was taken to the West Ham Hospital, where a scalp wound was dressed, and he was sent away, to be conveyed to the police-station, and charged with drunkenness. He was ultimately taken to the West Ham Infirmary, and died there about six the next evening. A post-mortem examination showed fracture of the skull, and a large blood clot as a result of hæmorrhage from divided vessels. It is hardly necessary to say that the jury were very severe upon the resident medical officer of the West Ham Hospital; and it is to be regretted that the coroner did not, according to the report, inform them of the absolute impossibility of discovering, in the case of a tipsy man who has fallen down, whether or not he may be the subject of intra-cranial bleeding from a small vessel. It is well known to all surgeons that the only safety in such cases is to detain the patient for a sufficient time to clear up all doubt. Every house surgeon, even if only for his own protection, would gladly take in all such doubtful cases were it not that the presence of a tipsy and often troublesome man in a ward may be very hurtful to other patients. Still, a patient about whose condition there can be a reasonable question ought not to be sent away if there is any possibility of retaining him, and all hospitals should take care to keep in reserve sufficient accommodation to meet common emergencies. It is much more a hospital's duty to be always ready than to be always full. We have often pointed out that it is no excuse to plead that the hospital is full. Every hospital ought to have accommodation, to wit, a few beds in a small room in which these suspicious cases may be retained under proper supervision, where they would be no nuisance in any way to the really sick or a danger to other inmates of the hospital. Several London and provincial hospitals which are well managed have such accommodation already, and it is the duty of the authorities to see that no hospital shall longer be without adequate provision of the kind referred to, so that all scandal may be avoided in future. Otherwise, we may expect that a British jury will very shortly bring in a verdict of manslaughter against the chairman or resident medical officer of any hospital which neglects this plain duty to the public.

### The Recrudescence of Plague in Bombay.

The very definite recrudescence of the plague which has taken place in Bombay during the past few weeks again directs attention to that wretched disease. The latest news states that the death-rate from plague has risen to upwards of 100 in the twenty-four hours, which is a most serious state of affairs. The tendency to a second outbreak, which seems almost part of the natural history of epidemics of plague, may, perhaps, be to some extent due to the return of emigrants who had fled the city during the prevalence of the disease, perhaps partly to the resumption of old habits and the reoccupation of plague-stricken houses, but in any case it is by no means peculiar to the present epidemic; the same thing having occurred at Canton and Hong Kong in recent years, as well as in some of the epidemics recorded in history. Besides its recrudescence in the city of Bombay, the wide diffusion of plague through the Presidency, and the virulence with which it has attacked some of the towns, such as Poona, must make us anxious as to its future progress, especially when we bear in mind the traditional connection between war and pestilence. Wars, especially in the East, cause large movements of population and give many opportunities for the wide distribution of disease, and it is impossible to regard without anxiety what is going on upon our frontier, in view of the great fatality of plague, when in former times it has obtained a footing in Persia and in those regions which have now become part of Southern Russia. We understand that the Indian Government has telegraphed to England for eight more medical men, two more medical women, and 26 more nurses, in addition to those sent out some time ago for temporary plague work; and the Viceroy adds that if the plague should extend still more will be required. It is becoming additionally apparent that the emergency can only be effectually met by the employment of European organisation.

